# Catestatin peptide impedes melanoma progression and drug resistance by reprogramming oncogenic signaling pathways

**DOI:** 10.1038/s41389-026-00628-y

**Published:** 2026-05-21

**Authors:** Satadeepa Kal, Suborno Jati, Kechun Tang, Nicholas J. G. Webster, Angelo Corti, Sushil K. Mahata

**Affiliations:** 1 Veterans Medical Research Foundation, San Diego, CA USA; 2 Department of Medicine, University of California, San Diego, CA USA; 3 Department of Neurosciences, University of California, San Diego, CA USA; 4 VA San Diego Healthcare System, San Diego, CA USA; 5 IRCCS San Raffaele Scientific Institute, San Raffaele Vita-Salute University, Milan, Italy

**Keywords:** Melanoma, Stress signalling

## Abstract

Melanoma remains one of the most aggressive and therapy-resistant cancers, underscoring the need for innovative therapeutic strategies. In our current study we report a peptide-based approach as potential therapeutic. Here we report for the first time the involvement of Catestatin (CST) peptide in carcinogenesis, with melanoma identified as unexplored and therapeutically relevant context. The expression and role of CST, a Chromogranin A (CgA)-derived peptide with immunomodulatory and reparative properties in skin injury, led us to examine its connection to melanoma. Analysis of human melanoma tissues revealed that CST expression decreases with advancing disease stage, suggesting a potential tumor-suppressive function. Restoration of CST in patient-derived melanoma cells and established melanoma cell lines (A375, B16F10, and SKMEL28) induced apoptosis and suppressed proliferation and migratory capacity, while normal skin fibroblasts remained unaffected, indicating tumor-selective activity. In vivo, CST administration significantly reduced tumor growth and tumor weight in the B16F10 melanoma mouse model, with no detectable systemic toxicity. Transcriptomic profiling of CST-treated melanoma cells and tumors revealed downregulation of pathways involved in hypoxia signaling, extracellular-matrix remodeling, epithelial-to-mesenchymal transition (EMT), and stress-adaptive responses, key drivers of melanoma invasion and progression. Consistent with these findings, CST suppressed several mediators of tumor progression. CST also reduced the viability and migration of Vemurafenib-resistant A375 cells, accompanied by the downregulation of multiple resistance-associated genes. Together, these findings establish catestatin as a novel regulator of melanoma growth and therapeutic resistance and provide a mechanistic rationale for the development of CST-based peptide therapeutics targeting both treatment-naive and drug-resistant melanoma.

**Figure:**
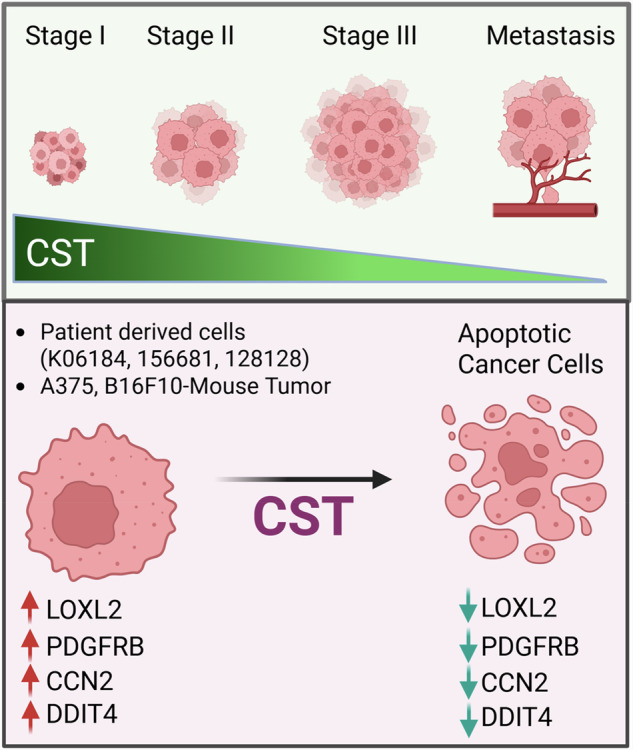
Catestatin Suppresses Tumor Progression and Metastasis by Inducing Apoptosis and Inhibiting Pro-tumorigenic Signaling. Catestatin (CST), a Chromogranin A–derived peptide, exhibits potent anti-tumor activity by suppressing cancer progression across multiple stages. CST levels decline with advancing tumor stage, suggesting a loss of endogenous tumor restraint during progression to metastasis. In patient-derived melanoma cells and tumor models (A375 and B16F10), CST treatment induces apoptotic cell death. Mechanistically, CST downregulates key pro-tumorigenic and pro-fibrotic signaling molecules, including LOXL2, PDGFRB, CCN2, and DDIT4, which are associated with extracellular-matrix remodeling, growth factor signaling, and cellular stress adaptation. These findings identify CST as a novel regulator of tumor survival and metastatic potential, supporting its therapeutic potential as a peptide-based anti-cancer agent.

Catestatin Suppresses Tumor Progression and Metastasis by Inducing Apoptosis and Inhibiting Pro-tumorigenic Signaling. Catestatin (CST), a Chromogranin A–derived peptide, exhibits potent anti-tumor activity by suppressing cancer progression across multiple stages. CST levels decline with advancing tumor stage, suggesting a loss of endogenous tumor restraint during progression to metastasis. In patient-derived melanoma cells and tumor models (A375 and B16F10), CST treatment induces apoptotic cell death. Mechanistically, CST downregulates key pro-tumorigenic and pro-fibrotic signaling molecules, including LOXL2, PDGFRB, CCN2, and DDIT4, which are associated with extracellular-matrix remodeling, growth factor signaling, and cellular stress adaptation. These findings identify CST as a novel regulator of tumor survival and metastatic potential, supporting its therapeutic potential as a peptide-based anti-cancer agent.

## Introduction

Melanoma is an escalating cancer worldwide, with incidences rising faster than any other solid tumor over recent decades [1]. Although melanoma represents a small fraction of skin cancers, it accounts for over 80% of skin cancer–related deaths owing to its aggressive metastatic potential and resistance to therapy [1]. The advent of targeted therapies - particularly BRAF and MEK inhibitors - and immune checkpoint blockade has substantially improved outcomes for patients with advanced disease [2, 3]. Small-molecule inhibitors Dabrafenib and Vemurafenib, which selectively target *BRAF^V600E* mutations, are now the standard of care for a majority of melanoma patients [3, 4], whereas those harboring *KIT* mutations benefit from Imatinib [5]. Moreover, the advent of immune checkpoint inhibitors has revolutionized melanoma management, extending median survival from approximately six months to nearly six years [6].

Despite breakthroughs, a multitude of patients either fail to respond or acquire resistance during treatment, resulting in relapse and mortality [7, 8]. These limitations highlight the urgent need for novel therapeutic approaches engaging mechanisms beyond the current ones.

Chromogranin A (CgA), a prohormone abundantly expressed in neuroendocrine tissues [9, 10], undergoes proteolytic processing, generating multiple biologically active peptides. Among them, Catestatin (CST; hCgA_352-372_) emerged as a multifunctional regulator of cardiovascular, metabolic, and immune homeostasis [11–15]. CST exerts protective effects, including anti-inflammatory [16, 17], anti-hypertensive [18–22], and anti-diabetic [16, 23] actions. It inhibits catecholamine release [24–26], regulates endothelial proliferation and migration [17, 27, 28], and modulates macrophage and lymphocyte phenotypes [16], supporting its role as a systemic homeostatic peptide with therapeutic potential.

CST is expressed in human skin, with increased levels following injury [29], and exerts functional activity in keratinocytes [30]. CST-like immunoreactivity has been reported in rodent skin and sensory ganglia [31], suggesting a conserved cutaneous role. However, its relevance in oncogenic progression has not been explored.

In the present study, we identify CST as a previously unrecognized regulator of melanoma progression in both human and mouse models. We demonstrate that CST expression decreases as melanoma progresses, and that exogenous CST administration markedly suppresses melanoma cell viability, proliferation, and migration across patient-derived and established cell lines. In syngeneic B16F10 tumor models, CST administration significantly reduces tumor growth and enhances apoptosis, confirming its anti-tumorigenicity in vivo. Transcriptomic analyses reveal that CST downregulates key pathways associated with extracellular-matrix organization, hypoxia response, and collagen metabolism - hallmarks of melanoma invasion and metastasis. Notably, CST suppresses several resistance-associated genes, including *FGFR3, PDGFRB, ID1/2/3, SREBF1*, and *MITF*, in Vemurafenib-resistant A375 cells, suggesting a role in overcoming therapeutic resistance.

Collectively, our findings establish CST as a novel melanoma-regulatory peptide with multifaceted anti-tumor activity. By integrating clinical, molecular, and in vivo evidence, this study positions CST as a promising therapeutic candidate. Given its pleiotropic regulatory properties and favorable safety profile, CST may inaugurate a new class of peptide-based therapeutics that modulate oncogenic pathways to inhibit melanoma progression and resistance.

## Materials and methods

All methods were performed in accordance with the relevant institutional guidelines and regulations.

### Cell culture

Mouse embryonic fibroblast (MEF), human skin fibroblast (CCD1076), human melanoma cell lines (A375, SK-MEL-28), and mouse melanoma cell line (B16F10) were obtained from the American Type Culture Collection (ATCC). Cells were maintained in DMEM supplemented with 10% fetal bovine serum (FBS), 100 U/mL penicillin, and 100 µg/mL streptomycin in a humidified incubator at 37 °C with 5% CO₂. Patient-derived melanoma cell lines (K06184, 156681, and 128128-mixed tumor cultures) were procured from the NCI Patient-Derived Models Repository (PDMR) and cultured following PDMR standard operating procedures in DMEM/F-12 containing 5% FBS, 0.4 µg/mL hydrocortisone, 0.01 µg/mL epidermal growth factor, 24 µg/mL adenine, 100 µg/mL penicillin-streptomycin, 2 mM L-glutamine, and 10 nM Y-27632 dihydrochloride on Matrigel-coated plates. Details of patient-derived melanoma cells can be obtained from https://pdmdb.cancer.gov/web/apex/f?p = 101:41 and a short description from Supplementary Table 1. All cells were checked for mycoplasma using Universal mycoplasma detection kit from ATCC (Catalog #30-1012 K).

### Human tissue samples

Commercially available human melanoma tissue microarrays (catalog nos. Me482A and Me551) were purchased from TissueArray.com for immunohistochemical analyses.

### Peptide treatment

Catestatin (CST; sequence SSMKLSFRARAYGFRGPGPQL) was synthesized by GenScript and dissolved in 0.9% saline. Cells were treated with CST at concentrations of 0.5–10 µM as indicated. For in vivo studies, CST was administered intraperitoneally at 10 mg/kg body weight, three times per week (Monday, Wednesday, Friday).

### CST cellular uptake assay

Biotinylated CST synthesized from GenScript was added to A375 cells; 5 µM for 45 min. Cells were fixed in paraformaldehyde and AF488-tagged Streptavidin (catalog #S32354) and Hoechst was added in permeabilized cells for 1 h. Cells were washed in PBS and mounted with Fluoromount G (Thermo Scientific) and observed in 60X oil immersion lens in a Nikon AXR Confocal, NSPARC super resolution microscope in UCSD core.

### Bioinformatic analyses

Expression of CCN2, LOXL2, DDIT4, and PDGFRB in primary and metastatic skin cutaneous melanoma samples was analyzed using the UALCAN database (http://ualcan.path.uab.edu) [32].

### Caspase assay

Cells (1 × 10⁴ per well) were seeded in 96-well white plates and treated with or without CST for 48 h (A375, SK-MEL-28, B16-F10) or 120 h (patient-derived lines K06184, 128128, 156681). Caspase-3/7 activity was measured using the Caspase-Glo^®^ 3/7 Assay System (Promega-G8090) following the manufacturer’s protocol. Luminescence was recorded using a Varioskan LUX microplate reader (Thermo Fisher Scientific).

### Live/dead viability assay

Cells treated with CST or vehicle for 120 h were incubated with 2 µM calcein AM and 4 µM ethidium homodimer III (Biotium Viability/Cytotoxicity Assay Kit #30002) for 30 min at room temperature in the dark. After washing, cells were imaged under FITC and Texas Red^®^ channels using a Keyence fluorescence microscope (20×).

### Cell viability assay

Cells (1 × 10⁴ per well) were plated in 96-well plates and treated with CST (0.5–10 µM) for 72–120 h. Viability was determined using the CCK-8 reagent (APExBIO-K1018). Absorbance was measured at 450 nm using a Varioskan LUX reader.

### Colony-formation assay

A375, SKMEL28, and B16F10 cells (1 × 10³ per well) were treated with or without CST according to the experimental design and cultured for 15 days. Colonies were fixed in methanol for 20 min, stained with 0.1% crystal violet, and photographed.

### Transwell migration assay

Equal number of cells were seeded in serum-free medium in transwell inserts (8 µm pore size) and were treated with CST or control for 48 h. The lower chamber contained complete medium. After 48 h, non-migrated cells were removed, membranes were fixed, stained with crystal violet, and imaged using a Keyence brightfield microscope (20×) as described previously [33].

### Wound-healing assay

A375 cells at approximately 50% confluency was scratched with a sterile pipette tip, followed by CST or control treatment. Images were captured at 0 and 24 h using brightfield microscopy (20×).

### RNA sequencing

Total RNA from mice tumor and A375 cell line was isolated using RNeasy miniprep Kit (Qiagen). RNA was quantified by Nanodrop spectrophotometer, and integrity was evaluated by Tapestation (Agilent). Complementary DNA library was prepared using 400 ng of RNA using mRNA HyperPrep Kit (KAPA) according to manufacturer’s protocol with Unique Dual-Indexed adapters (KAPA). The cDNA library was amplified and assessed by Qubit2.0 (Thermo Fisher Scientific). The libraries were then pooled, and sequencing was done in NovaSeq X Plus 10B (Illumina) in UCSD IGM core [34].

### RNA sequencing analysis

Raw RNA-seq reads were quantified using Salmon (v1.10.3) in quasi-mapping mode against the *Homo sapiens* or *Mus musculus* reference transcriptome (Human: Ensembl Release GRCh38.115, Mouse: Ensembl Release GRCm39.115). Transcript-level abundance estimates (quant.sf files) were generated for each sample. Transcript-to-gene mapping was obtained from the relevant Ensembl GTF annotation file using the GenomicFeatures and AnnotationDbi R packages. Transcript quantifications were imported and summarized at the gene level with tximport (v1.36.1), setting ignoreTxVersion = TRUE. The resulting gene-level count matrix served as the basis for further analysis.

#### Differential expression analysis (DESeq2)

Gene expression differences between treatment groups were assessed using DESeq2 (v1.48.1) in R. The workflow included normalization with size factors, dispersion estimation, and Wald tests, following the default DESeq2 procedures. Genes with an adjusted *p*-value (Benjamini - Hochberg FDR) < 0.05 and |log₂-fold change | > 1 were considered significantly differentially expressed.

#### Annotation and visualization

Ensembl gene IDs were mapped to gene symbols using the org.Hs.eg.db (v3.21.0, for Human) or org.Mm.eg.db (v3.21.0, for Mouse) annotation packages. Volcano plots, generated with ggplot2 (v4.0.0), highlighted significantly up- and downregulated genes. All analyses were based on DESeq2 results. Gene expression heatmaps were created using the Complex Heatmap package (v2.24.1) in R, based on Log2-transformed normalized counts. GO Biological Process Enrichment analysis of the up- and downregulated genes was conducted with clusterProfiler (v4.16.0) and shown as bar plots. Common genes between mouse and human transcriptomic analyses were identified using the VennDiagram package (v1.7.3) in R.

### Generation of vemurafenib-resistant cells

Vemurafenib-resistant A375 cells were generated following established protocol. Parental cells were treated with 100 nM Vemurafenib (SelleckChem #S1267) for 2 weeks, followed by stepwise doubling of drug concentration every 2 weeks up to 3.2 µM. Surviving cells in maintenance culture containing 2 µM drug were used for downstream studies.

### Mouse tumor model

Age-matched male and female *C57BL/6* mice (7–9 weeks, *n* = 9) were subcutaneously injected with 5 × 10⁴ B16-F10 cells in 100 µL PBS. Ten days post-injection, CST (10 mg/kg) was administered intraperitoneally three times weekly. Tumor dimensions were measured with calipers, and volume was calculated using the formula *V* = (length × width²)/2. Mice were euthanized 22 days post-inoculation by inhalational isoflurane, that follow the institutional IACUC protocol and AVMA guideline for Euthanasia of animals (2020). Animals were kept in an induction chamber containing 3–5% isoflurane in oxygen, which had a flow rate of 1–2 L/min. Animals were continuously monitored till the loss of righting reflex and response to toe pinch was absent, thus confirming a surgical plane of anesthesia. Tissues were collected when the animals went into deep anesthesia and euthanasia was finally completely by exsanguination. All procedures were approved by the IACUC of UCSD and the VA San Diego Healthcare System and conformed to NIH guidelines.

### Immunohistochemistry and histology

Fixed tumors were paraffin-embedded and sectioned (5 µm). Hematoxylin and eosin staining was performed at the La Jolla Institute for Immunology. Immunohistochemistry was carried out using the Super Sensitive™ M Polymer-HRP Kit (Biogenex). After deparaffinization, antigen retrieval was performed using Histo VT (Nacalai) at 90 °C for 20 min. Sections were incubated overnight at 4 °C with anti–Ki-67 (Abcam-16667), anti-CCN2, anti-LOXL2, anti-DDIT4 (Proteintech-25474-1-AP; 15232-1-AP; 10638-1-AP) anti-PDGFRB (Cell Signaling Technology-3169T) or anti-CST (gift from Angelo Corti) at a 1:100 dilution. Detection was performed with DAB and counterstained with hematoxylin. Slides were dehydrated and mounted in DPX. Images were captured using Keyence brightfield microscopy (20×). Images were converted to 8-bit grayscale, and identical threshold parameters were applied to both control and experimental images. The area fraction was calculated as the percentage of pixels that exceeded the threshold within the defined region of interest (ROI). Quantification was performed using Fiji (ImageJ), which computes the area fraction as the ratio of the threshold-positive area to the total ROI area, expressed as a percentage.

### TUNEL assay

Apoptotic nuclei were detected in tumor sections using the CF^®^594 TUNEL Assay Kit (Biotium #30064). After deparaffinization and rehydration, sections were permeabilized with 20 µg/mL Proteinase K for 1 h at 37 °C, incubated with TUNEL reaction mix for 2 h, counterstained with Hoechst, and mounted in antifade medium. Fluorescence images were obtained using a Keyence microscope.

### Phase-contrast microscopy

Morphological alterations in CST-treated A375 and Vemurafenib-resistant A375 cells were assessed using Keyence phase-contrast microscopy.

### Western blotting

Equal amounts (10 ng) of purified Chromogranin A (CgA) protein and Catestatin (CST) peptide were subjected to immunoblotting with antibodies against CgA (Thermo Fisher Scientific, MA1-3093) or CST (CgA_352-372_). For tissue lysates from wild-type and CST-knockout (CST-KO) mice [22], 10 µg of total protein was analyzed by immunoblotting using an anti-CST antibody. Equal protein amounts (10 µg) from tumor lysates prepared in RIPA buffer containing protease inhibitors (Thermo Fisher) were subjected to western blotting and incubated with antibodies against caspase-3 (Proteintech-19677-1-AP), β-actin (Cell Signaling Technology #4970S), LOXL2, CCN2, DDIT4, GAPDH, PDGFRB (Proteintech 10638-1-AP; 15232-1-AP; 25474-1-AP, 60004-1-Ig, Thermo #MA 515143). For the A375 cell line, an equal amount (15 µg) of con and CST-treated lysates was subjected to western blotting. They were probed using antibodies against DDIT4, LOXL2, CCN2, and caspase-3. Equal amounts (15 µg) of protein lysates from control and CST-treated A375 vemurafenib-resistant cells were analyzed by immunoblotting using antibodies against ID1, ID3, TWIST1, SPARC, and GAPDH (Proteintech; 18475-1-AP, 10389-1-AP, 25465-1-AP, 15274-1-AP, 60004-1-Ig). HRP-conjugated secondary antibodies (Cell Signaling Technology 7076S,7074S) were used, and signals were detected by chemiluminescence. Densitometric analyses were performed using BioRad Image Lab software.

### In vitro plasma stability and in vivo plasma pharmacokinetics

Plasma stability was determined in 5 µg CST/ml spiked mouse (C57BL/6) plasma (50 µl) and taking aliquots at specific times over a 24-h period. Each aliquot (50 µl) was mixed with 150 µl methanol, vortexed, spun, removed the supernatant and reconstituted the pellet with 150 µl 10% formic acid in water. The sample was vortexed and spun, and a 100 µl aliquot was taken, diluted 1:1 with 10 mM ammonium bicarbonate, and analyzed by LC-MS/MS. Using the remaining vs. time dataset, we performed a first-order decay analysis by fitting a linear regression to the natural log-transformed percent remaining values (excluding 0% and *t* = 0 anchor point) to plot the graph.

For in vivo plasma pharmacokinetics, male C57BL/6 mouse received a 5 mg/kg intraperitoneal dose of CST. Blood samples (100 µl) were obtained at 0, 0.25, 0.5, 1, 2, 4, 8, and 24 h via tail vein bleeds. Plasma concentrations of CST were determined by LC-MS/MS methods as described above.

### Real-time PCR

Total RNA was extracted from control and CST-treated vemurafenib-resistant A375 cells using TRIzol reagent according to the manufacturer’s instructions. Briefly, 500 ng of total RNA was reverse-transcribed into cDNA using Thermo Maxima™ H Minus cDNA Synthesis Master Mix (catalog #M1661). Quantitative real-time PCR (qRT–PCR) was performed to assess the expression of Actin, ID1, ID3, TWIST1, and SPARC using gene-specific primers (listed in Supplementary Table 3). Relative gene expression was calculated using the 2^−ΔΔCt method, and data were plotted using GraphPad Prism 10.

### Catestatin antibody validation

The rabbit polyclonal antiserum VFA/09-C2 was produced by PRIMM (Italy) by immunizing a rabbit with the peptide CSSMKLSFRARAYGFRGPGPQL (corresponding to Cys-CgA_352–372_) conjugated to keyhole limpet hemocyanin (KLH). The specificity of the antiserum was assessed by ELISA using microtiter plates coated with various fragments of Chromogranin A (CgA) (CgA_1–439_, CgA_1–400_, CgA_1–394_, CgA_1–373_, CgA_1–372_, CgA_1–338_, CgA_352–373_, CgA_352–372_, CgA_367–373_, CgA_367–372_, and CgA_1–78_ [vasostatin]), prepared by recombinant DNA technology or chemical synthesis as previously described [35]. For the ELISA assay, 96-well polystyrene microtiter plates were coated with polypeptides (10 µg/ml in phosphate-buffered saline; PBS, 0.15 M NaCl, 0.015 M sodium phosphate, pH 7.3; 50 µl per well) and incubated overnight at 4 °C. Plates were washed three times with PBS and blocked with 3% bovine serum albumin (BSA) in PBS (200 µl per well) for 2 h at room temperature. After washing with PBS, serial dilutions of rabbit antiserum prepared in PBS containing 0.5% BSA, 2.5% normal goat serum, and 0.05% Tween-20 (PBS-NT) were added to each well (50 µl per well) and incubated for 2 h at room temperature (RT), and washed again with PBS. Different dilutions of rabbit antiserum in PBS containing 0.5% BSA, 2.5% normal goat serum and 0.05% Tween 20 (PBS-NT), were then added to each well (50 µl/well) and left to incubate for 1.5 h at RT. Plates were washed eight times with PBS containing 0.05% Tween-20 (PBS-T) and incubated with goat anti-rabbit IgG conjugated to horseradish peroxidase (GAR-HRP, Sigma; 1:1000 dilution in PBS-NT) for 1 h at room temperature. Following washing with PBS-T, the HRP chromogenic substrate o-phenylenediamine (Sigma) was added, and the reaction was allowed to proceed for 20 min. The reaction was terminated by adding 70 µl per well of 10% sulfuric acid. Absorbance was measured at 490 nm using an ELISA plate reader.

### Statistical analyses

Data were analyzed using GraphPad Prism 10. Two-tailed Welch’s *t*-test was used for single-variable comparisons, and one-way or two-way ANOVA for multivariate datasets (e.g., concentration and time). Statistical significance was defined as *p ≤ 0.05, **p ≤ 0.01, ***p ≤ *0.001*, ***p ≤ *0.0001* and non-significant as NS.

## Results

### Catestatin levels decline with advancing melanoma and exhibit anti-proliferative effects on patient-derived melanoma cells

**Fig. 1:**
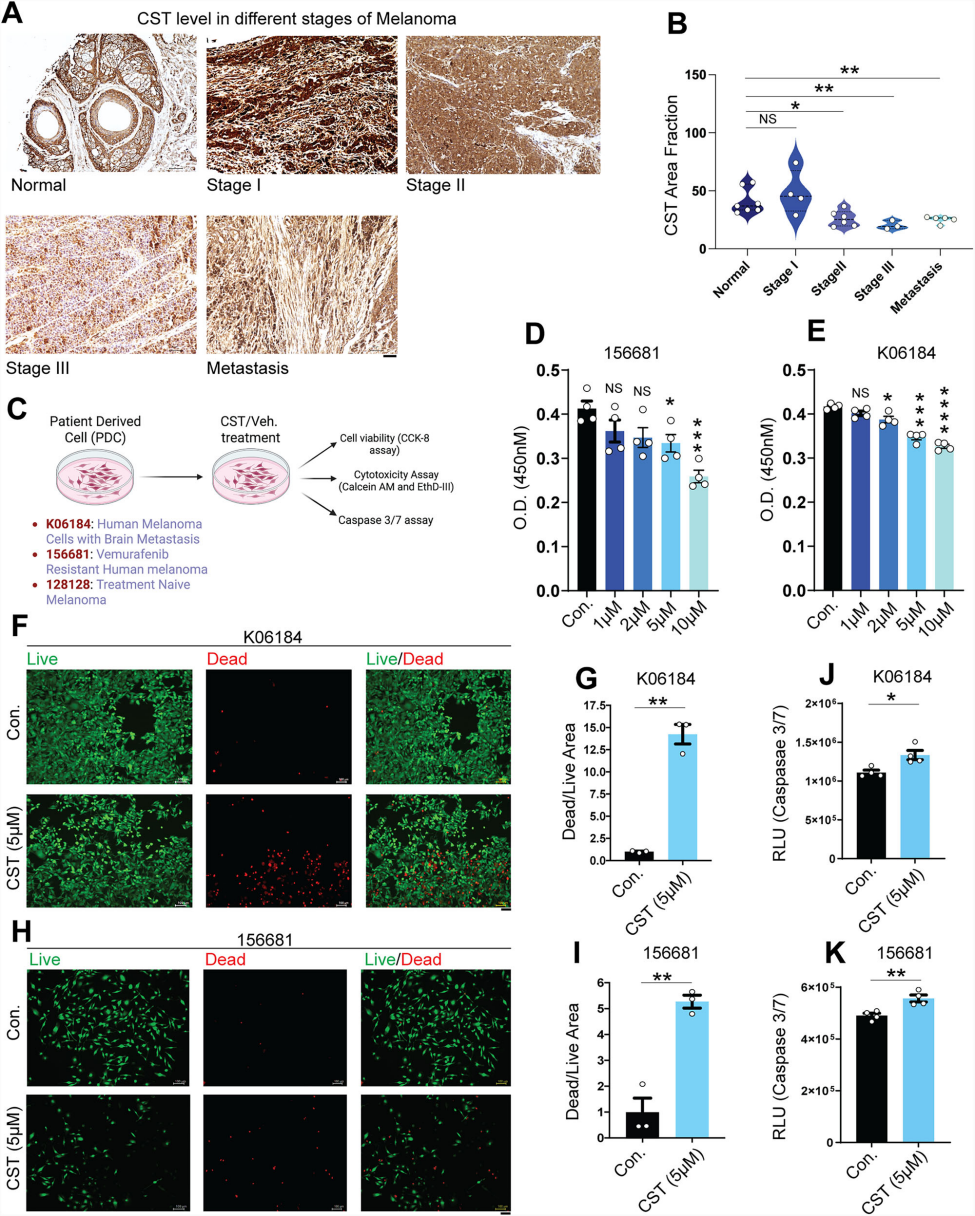
Decline in endogenous CST levels in human melanoma samples and melanoma cell proliferation inhibition upon extraneous CST treatment. **A** Representative immunohistochemistry images showing levels of Catestatin (CST) in different melanoma stages (normal, stage I, stage II, stage III, and metastasis) in human samples from TissueArray.com. **B** Quantification of the immunoreactive CST as area fraction as determined by Fiji3 software. **C** Schematic for experimental setup on melanoma patient-derived cells (K06184, 156681, and 128128). **D**, **E** Cell viability assay performed on patient-derived cell 156681 and K06184 for 120 h in response to different concentrations of CST (1 µM, 2 µM, 5 µM, and 10 µM as compared to vehicle control (*n* = 4). **F**, **H** Live and dead cell imaging of mammalian cells microscopy showing calcein AM (green) stained live cells and EthD-III (red) stained dead cells upon CST treatment versus control in K06184 and 156681 cells. Images were captured in a Keyence microscope at a magnification of 10X. Scale bar for imaging is 100 µm. **G**, **I** Dead/Live area fraction measured upon CST treatment (5 µM) in K06184 and 56681 cells (*n* = 3). **J**, **K** Caspase 3/7 assay done in CST-treated and untreated K06184 and 156681 cells. (*n* = 4). Data were presented as Mean ± SEM. Statistical analyses were done using one-way ANOVA (D&E) and Welch’s *t*-test (**B**, **G**, **J**, **I**, **K**). *p ≤ 0.05, **p ≤ 0.01, ***p ≤ 0.001, ****p ≤ 0.0001.

CST has been detected in human skin [29] and keratinocytes [30] as well as in rodent skin and ganglia [31], but its presence in melanoma patient samples remained unassessed. To address this, we analyzed CST protein expression across progressive stages of melanoma using tissue microarray slides (TissueArray.com). Immunohistochemical (IHC) analysis revealed a marked decline in CST expression with advancing melanoma stages, whereas normal skin and stage I melanoma tissues retained high CST levels (Fig. 1A). Quantitative analysis confirmed a significant reduction in CST staining intensity as melanoma progressed (Fig. 1B). The specificity of the CST antibody was validated using multiple complementary approaches. ELISA analysis (Supplementary Fig. S1A, B) demonstrated that the antiserum robustly recognized CgA_1-373_, CgA_1-372_, CgA_352-373_, and CgA_352-372_ fragments, but not CgA_1-439_, CgA_1-400_, CgA_1-338_, CgA_367-373_, CgA_367-372_, or CgA_1-78_ (vasostatin). These results localize the antibody epitope to residues 352–366 within the CST region. Notably, the absence of binding to full-length CgA (CgA_1-439_) and other larger fragments suggests that this epitope is structurally masked within intact CgA and becomes accessible only following proteolytic cleavage at residues 372 or 373. Consistent with this interpretation, immunoblotting using purified proteins and tissue lysates from wild-type and CST-KO mice confirmed that the CST antibody does not cross-react with full-length CgA (Supplementary Fig. S1C, D), demonstrating high specificity for the processed CST peptide.

To determine whether CST administration affects melanoma cell viability, we used patient-derived melanoma cell lines K06184, 156681, and 128128 (characteristics mentioned in Supplementary Table 1), which were mixed tumor cultures from the NCI-PDMR repository, and normal human dermal fibroblasts (CCD1076). A schematic of the experimental plan with the patient-derived cell lines is mentioned (Fig. 1C). CST treatment (1 µM, 2 µM, 5 µM, 10 µM) for 120 h caused dose-dependent reduction in melanoma cell viability (Fig. 1D, E and Supplementary Fig. S1F), whereas normal fibroblasts remained unaffected even at the highest concentration (Supplementary Fig. S1E). Viability assays showed elevated dead cells (EthD-III, red) relative to live cells (Calcein AM, green) following CST treatment (Fig. 1F–H and Supplementary Fig. S1G). Quantification revealed a significantly elevated dead-to-live cell ratio (Fig. 1G–I and Supplementary Fig. S1H). Corroboration of these results by increased caspase-3/7 activity confirm induction of apoptosis (Fig. 1J, K and Supplementary Fig. S1I), and hint at a possible role of CST in curbing melanoma progression.

### Catestatin translocates to melanoma cells and reduces viability, proliferation, and migration in melanoma cell lines

**Fig. 2:**
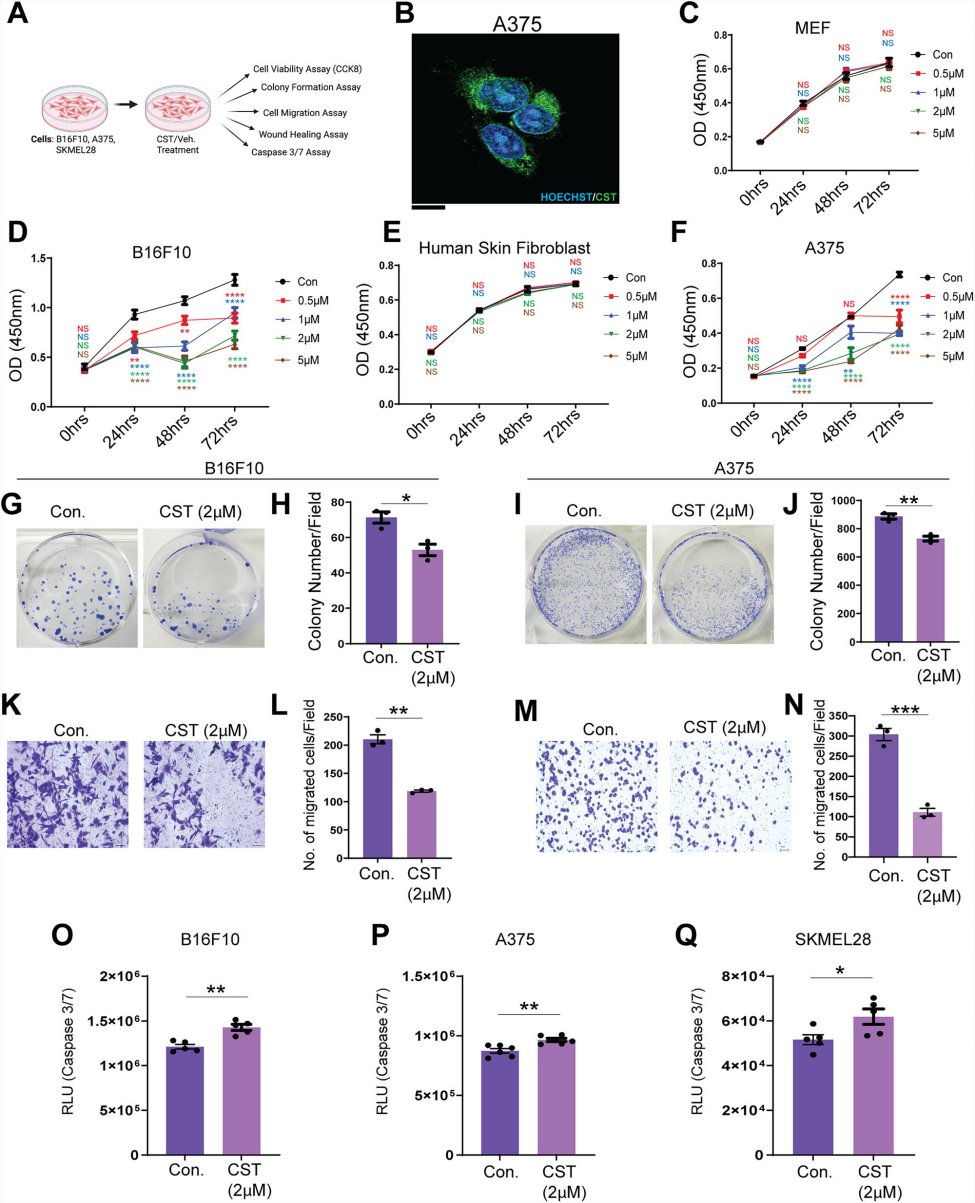
Melanoma progression inhibition upon CST treatment in mouse and human melanoma cell lines. **A** Schematic representation of the experimental setup on B16F10, A375 and SKMEL28 cell lines in control and CST-treated conditions. **B** Confocal imaging of cell penetration of biotinylated CST in A375 cells visualized by AF488-tagged streptavidin. The nuclei are stained with Hoechst. Magnification 60X. Scale bar-5 µm. **C**, **D** Cell viability of mouse embryonic fibroblast (MEF) and mouse melanoma cell line B16F10 by CCK-8 assay using different concentrations of CST (0.5 µM, 1 µM, 2 µM, and 5 µM) versus vehicle control for 0, 24, 48 and 72 h. **E**, **F** Cell viability by CCK-8 assay on normal human skin fibroblast and melanoma cell lines A375 in response to different concentrations of CST (0.5 µM, 1 µM, 2 µM, and 5 µM) for 0, 24, 48 and 72 h. For all cck-8 assay (*n* = 4). **G**, **H** Colony-formation assay and its quantitative analysis in B16F10 mouse melanoma cells. **I**, **J** Colony formation and its quantitative analysis in A375 human melanoma cell (*n* = 3). **K**, **L** Transwell migration assay in CST versus vehicle-treated condition and its quantitative analysis in B16F10 cells. **M**, **N** Transwell migration assay in CST versus vehicle-treated condition along with quantitative analysis in A375 human melanoma cells (*n* = 3). These brightfield images were captured in a Keyence microscope at 10X magnification. Scale bar: 100 µm. **O**–**Q** Caspase3/7 Assay in CST versus vehicle-treated condition in B16F10, A375 and SKMEL28 cells (*n* = 5). Data were presented as Mean ± SEM. Two-way ANOVA followed by Dunnett’s multiple comparison test was used to analyze cell viability assay data. Welch’s *t*-test was used to analyze colony formation, transwell migration and Caspase3/7 assay data. *p ≤ 0.05, **p ≤ 0.01, ***p ≤ 0.001, and ****p ≤ 0.0001.

To further our preliminary observations on CST’s melanoma-suppressive role, we performed experiments in established mouse and human melanoma cell lines and their normal counterparts, as shown in schematic (Fig. 2A). We treated human melanoma cell line A375 with biotinylated CST for 45 min and added AF488-tagged streptavidin to visualize CST localization. Confocal imaging revealed CST uptake into A375 cells (Fig. 2B). We next performed a cell viability assay in mouse melanoma cell line B16F10 and the human melanoma cell lines A375 and SKMEL28 with increasing concentration of CST (0.5 µM, 1 µM, 2 µM, and 5 µM) for 72 h. While there was no significant reduction in cell viability of normal mouse fibroblast or human skin fibroblast cell lines (Fig. 2C, E), we recorded a decline in cell viability of the melanoma cell lines (Fig. 2D, F and Supplementary Fig. S2A), thus indicating that CST targets the cancer cells, sparing the normal ones. Based on the maximal effects, a concentration of 2 µM was used for subsequent assays.

Phase-contrast microscopy revealed increased cell death in A375 cells treated with CST for 24 h (Supplementary Fig. S2B). Colony-formation assays showed that CST ablated clonogenic potential of both human and mouse melanoma cells (Fig. 2G–J; Supplementary Fig. S2C, D). Because cell migration is a hallmark of metastatic potential, we evaluated migration using transwell and wound-healing assays. CST significantly impaired migration in all melanoma lines compared with controls (Fig. 2K–N and Supplementary Fig. S2E–H). Increased caspase-3/7 activity in CST-treated melanoma cells (Fig. 2O–Q) further confirmed apoptotic induction. Collectively, CST inhibits melanoma cell survival, proliferation, and metastatic potential.

### Catestatin reduces melanoma tumor burden in vivo

**Fig. 3:**
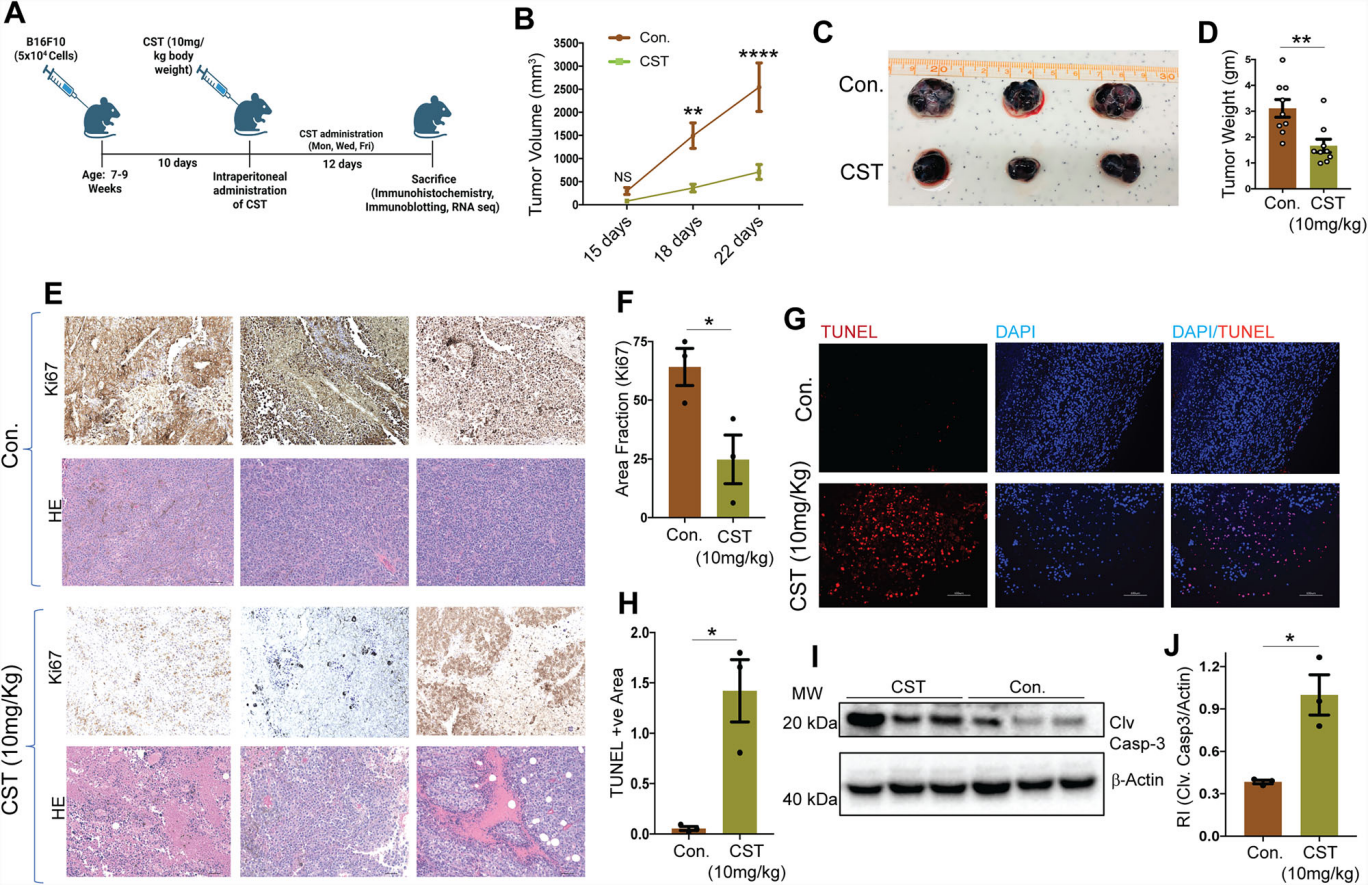
In vivo melanoma tumor is reduced upon CST treatment. **A** Schematic showing injection of 5 * 10^^4^ B16F10 cells to 7–9 weeks old C57BL/6 mice and the administration of CST (10 mg/kg) thrice a week till sacrifice. **B** Tumor growth kinetics in B16F10-derived tumors treated with CST (10 mg/kg) and vehicular control (*n* = 9). **C** Post-harvesting images of Control and CST-treated tumors. **D** Bar graph showing changes in tumor weight in response to control or treatment with CST (*n* = 9). **E** Immunohistochemistry images showing levels of proliferation marker Ki-67 in Control and CST-treated mice tumors. Hematoxylin and eosin staining reveal the tissue structures of the tumors. Images were captured in brightfield in Keyence microscope at a magnification of 20×. Scale bar: 50 µm. **F** Bar graph showing area fraction of Ki-67 staining in control and CST-treated tumors (*n* = 3). **G** TUNEL assay performed on control and CST-treated tumor sections. The TUNEL-positive cells stain red, and the nuclei stain blue. Images were captured in a Keyence microscope at a magnification of 10X. Scale bar: 100 µm. **H** The TUNEL-positive area of different tumors is represented in the graph (*n* = 3). (**I**) Representative western blot of cleaved caspase-3 as compared to actin levels in mice after treatments with control or CST-treated tumors. **J** Bar graph showing densitometric analysis of (**I**) (*n* = 3). Data were presented as Mean ± SEM and analyzed by 2-way ANOVA followed by Tukey’s multiple comparison test (tumor growth kinetics) or by Welch’s *t*-test. *p ≤ 0.05, **p ≤ 0.01, and ****p ≤ 0.001.

To assess CST efficacy in vivo, C57BL/6 mice were subcutaneously injected with 5 × 10⁴ B16F10 cells and treated intraperitoneally with CST (10 mg/kg, three times per week) (Fig. 3A). Our in vitro plasma stability results revealed that percent remaining declined mono-exponentially over 24 h. Linear regression of In (% remaining) versus time yielded a slope of –0.1246 h^−^¹ and an intercept of 4.0108, corresponding to an apparent first-order rate constant *k* = 0.1246 h^−1^ and a half-life *t*_1/2_ = 5.56 h. Model fit was strong (R^2^ = 0.971). These results indicate moderate plasma stability for the peptide under the assay conditions, with ~50% of intact material remaining at ~5.6 h (Supplementary Fig. S3A). We found the following in vivo plasma pharmacokinetic values: *C*_max_ (maximum concentration): 160.3 ng/ml; *T*_max_ (time to reach Cmax): 0.25 h; *t*_1/2_ (half-life): 1.49 h (Supplementary Fig. S3B). CST treatment significantly suppressed tumor growth compared with vehicle controls (Fig. 3B) as revealed from the tumor volume kinetics. Tumor weight also decreased in CST-treated mice (Fig. 3C, D). No major changes in body weight or liver histology were observed (Supplementary Fig. S3C, D), indicating systemic tolerability. IHC staining revealed reduced Ki-67 expression, indicating diminished proliferation (Fig. 3E, F). Hematoxylin and eosin staining showed reduced cellular density (Fig. 3E), and TUNEL assay demonstrated increased apoptotic nuclei in CST-treated tumors (Fig. 3G, H). Western blot analysis confirmed elevated cleaved caspase-3 levels (Fig. 3I, J). Intratumoral injection of CST also revealed decreased tumor size and weight (Supplementary Fig. 3E, F). Thus, CST treatment significantly curtails melanoma growth in vivo without observable toxicity.

### Molecular mechanisms underlying CST-mediated melanoma regression

**Fig. 4:**
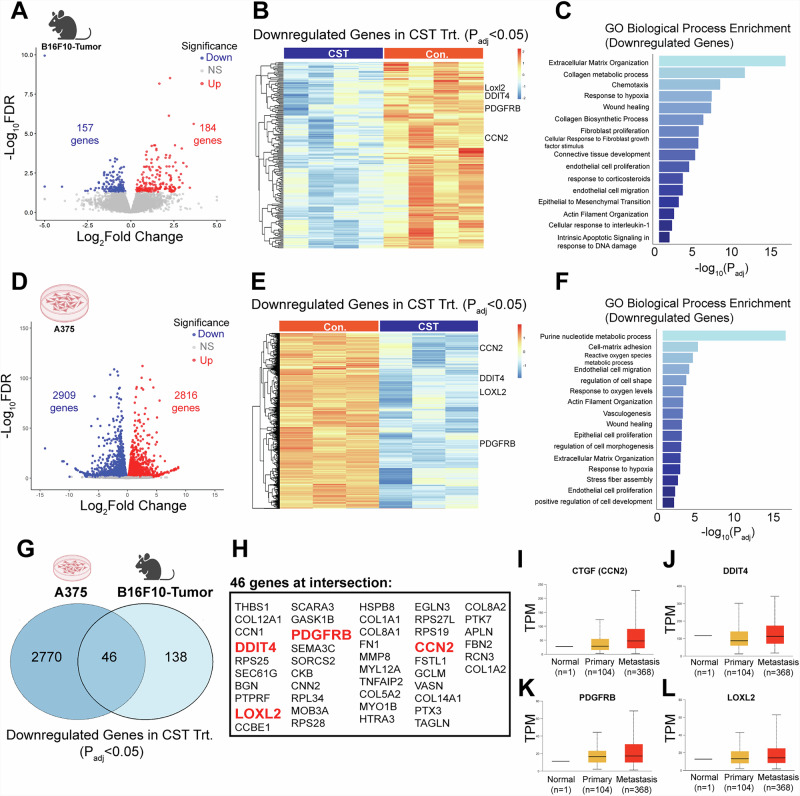
Molecular mechanisms driving CST function in mouse tumor and human melanoma cell line. **A** Volcano plot of differential regulation of genes upon CST treatment. **B** Heat map showing all downregulated genes with *p*adj < 0.05 with emphasis on CCN2, LOXL2, DDIT4, and PDGFRB genes (*n* = 4, each group) (**C**) Gene ontology (GO) analysis of enriched downregulated pathways emphasizing the previously mentioned genes. **D** Volcano plot of differentially expressed genes upon CST treatment in A375 human melanoma cell line. **E** Heatmap of downregulated genes with *p*adj < 0.05 (*n* = 3, each group) (**F**) GO analysis of enriched downregulated pathways upon CST treatment in A375 cells. **G** Venn Diagram showing common downregulated genes in the B16F10-derived mouse tumor and the A375 cell line. **H** List of the 46 common genes in the Venn Diagram with emphasis on *CCN2*, *LOXL2*, *DDIT4*, and *PDGFRB*. **I**–**L** Expression levels of *CCN2*, *DDIT4*, *PDGRB*, and LOXL2 from the TCGA database in primary melanoma vs metastasis as analysed in the UALCAN database. TPM- Transcripts per million.

To elucidate mechanisms of CST-induced tumor suppression, bulk RNA sequencing was performed on control and CST-treated B16F10 tumors. Differential expression analysis revealed widespread transcriptional modulation (Fig. 4A) with 184 upregulated and 157 downregulated genes upon CST treatment in vivo. Gene-ontology enrichment of upregulated (Supplementary Fig. S4A, B) and downregulated transcripts were mapped and when emphasized on the downregulated pathways they were linked to *extracellular-matrix (ECM) organization*, *collagen metabolism*, *response to hypoxia*, and *epithelial-to-mesenchymal transition (EMT)* (Fig. 4B, C)—processes that facilitate melanoma progression [36–38].

Similarly, transcriptomic analyses of CST-treated A375 cells showed 2816 upregulated and 2909 downregulated genes (Fig. 4D) The differentially expressed genes (Supplementary Fig. S4C, D) were mapped and the downregulated pathways related to *cell-substrate adhesion*, *reactive oxygen species metabolism*, and *hypoxia response* (Fig. 4E, F) were also observed in human melanoma A375 cell line. Comparative analysis of the mouse and human cell line datasets revealed 46 commonly downregulated genes (Fig. 4G, H), including *CCN2*(Cellular Communication Network factor 2)*, LOXL2* (lysyl oxidase-like-2), *DDIT4* (DNA damage-inducible transcript 4) and *PDGFRB* (Platelet-derived growth factor B), all previously implicated in melanoma aggressiveness [39–42]. Consistent with these findings, TCGA-SKCM analyses via UALCAN confirmed elevated expression of these genes in advanced melanoma stages (Fig. 4I–L, Supplementary Table 2). Together, these results suggest that CST suppresses melanoma by downregulating ECM remodeling, hypoxia, and EMT-associated signaling programs.

### CST treatment attenuates the expression of melanoma pro-survival genes *CCN2*, *LOXL2*, *DDIT4* and *PDGFRB* in mice melanoma tumor and human melanoma cell line

**Fig. 5:**
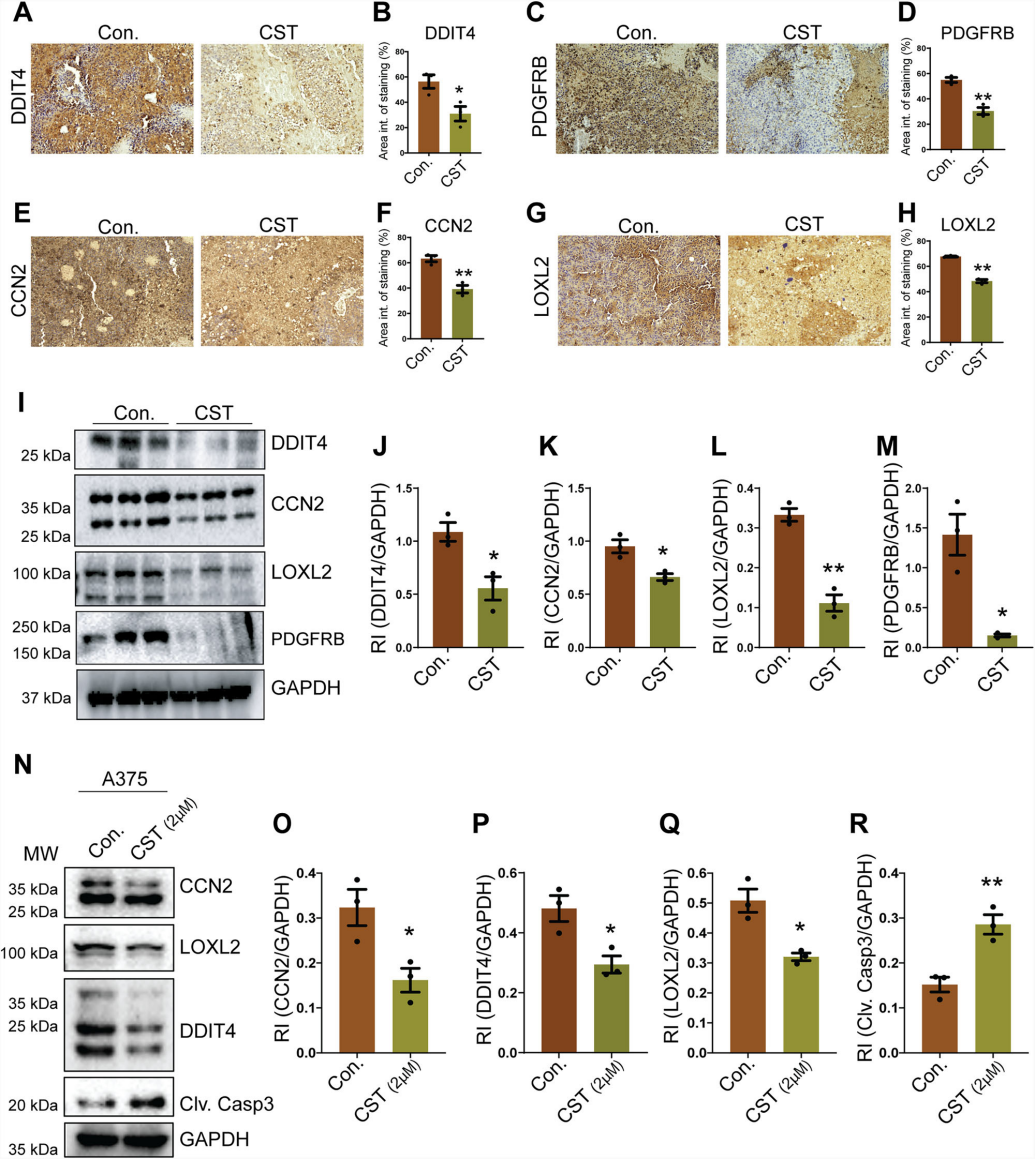
CST treatment declined expression of pro-survival proteins in the melanoma mouse tumor and the human melanoma cell line. **A** Immunostaining of control and CST-treated Formalin fixed paraffin embedded mice tumor tissue section for DDIT4 antigen. **B** Quantification of DDIT4 staining. **C** Immunostaining of PDGFRB in control and CST-treated mouse tumor sections. **D** Quantification of PDGFRB staining. **E**, **F** Immunostaining of Tumor sections for CCN2 antigen and its quantification. **G**, **H** Immunostaining and quantification of tumor tissue sections for LOXL2 antigen. **I**–**M** Western blot from control and CST-treated tumor tissue for DDIT4, CCN2, LOXL2, PDGFRB, GAPDH, and their quantification. **N** A375 human melanoma cell line treated with CST and control, and western blot of CCN2, LOXL2, DDIT4, and cleaved caspase 3. **O**–**R** Quantification of CCN2, DDIT4, LOXL2, and cleaved caspase3. All quantification graphs were performed on a sample size (*n* = 3) and analysed by Welch’s *t*-test. *p ≤ 0.05, **p ≤ 0.01, ***p ≤ 0.001, and ****p ≤ 0.0001.

The importance of genes *CCN2, LOXL2*, *DDIT4* and *PDGFRB* in melanoma progression is well established. CCN2, a connective tissue growth factor protein, is expressed abundantly in melanoma and aids in metastasis by allowing cellular movement through the extracellular matrix [42]. LOXL2 is known to be upregulated in clinical human melanomas compared to benign nevi and is often associated with metastasis and low survival of patients [43]. DDIT4, on the other hand, is a DNA damage-inducible protein and has elevated expression in several cancers, where it can promote angiogenesis and thus enhance cancer progression [43, 44]. PDGFRB plays a crucial role in melanoma, and its upregulation has often been associated with drug resistance [39, 45]. These genes are involved in forming new vasculature, respond to hypoxia, and directly and indirectly promote EMT, thereby worsening the prognosis. We further validated the expression of the proteins associated with these genes in B16F10-derived mouse melanoma tumor. Control and CST-treated tissue sections were immunostained for proteins DDIT4 (Fig. 5A, B), PDGFRB (Fig. 5C, D), CCN2 (Fig. 5E, F), LOXL2 (Fig. 5G, H) and a Western blot was also performed (Fig. 5I) and decreased expression levels of these proteins were observed in CST-treated tissue sections. Quantification of these protein levels in Con and CST-treated tumors revealed a significant reduction in protein expression levels (Fig. 5J–M). In the human melanoma cell line A375 treated with CST and its control, we observed diminished expression of CCN2, DDIT4, and LOXL2 proteins and increased cleaved caspase-3 levels (Fig. 5N–R), thereby implicating a pro-apoptotic role of CST administration in melanoma.

### CST suppresses Vemurafenib-resistant melanoma

**Fig. 6:**
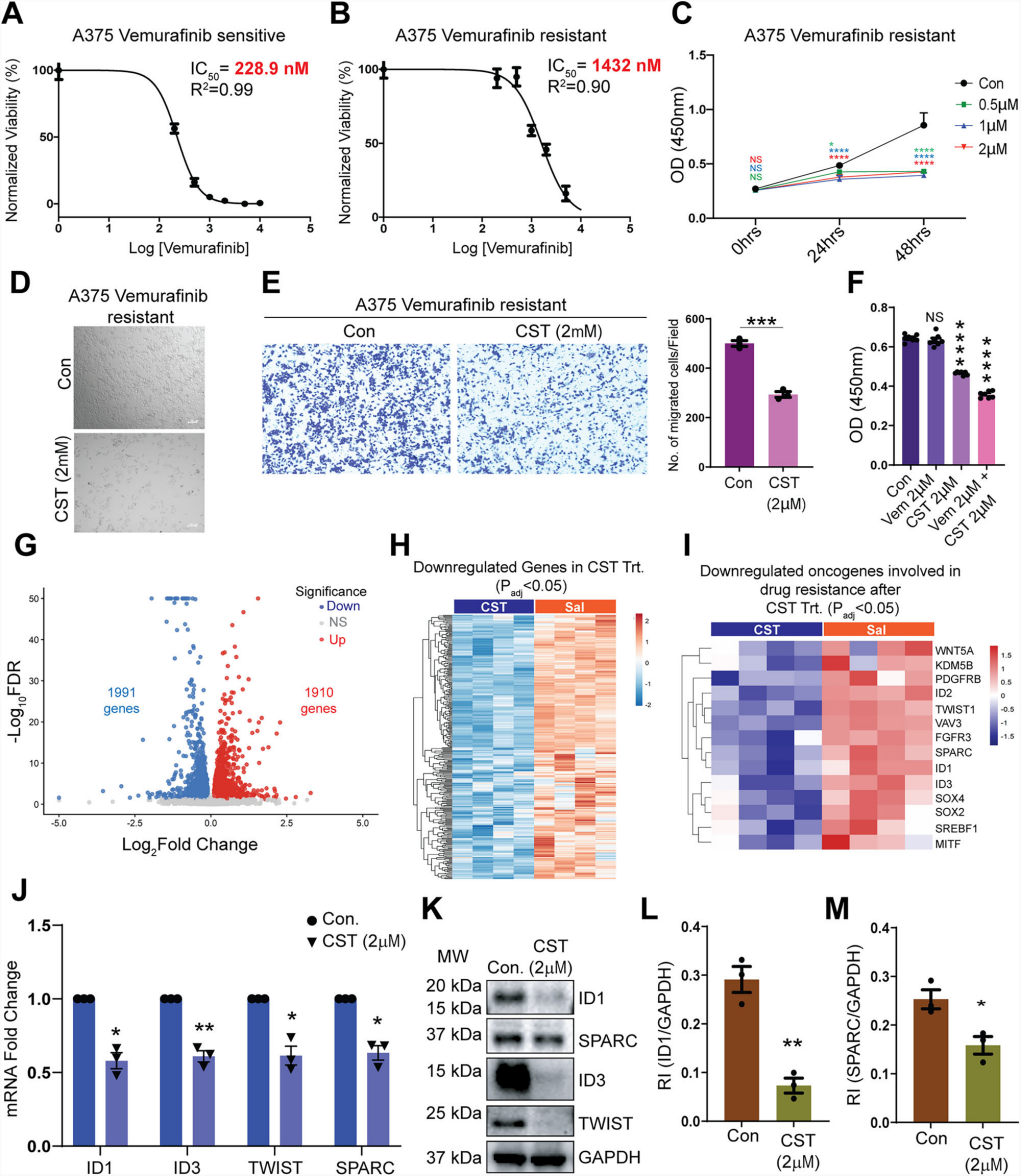
CST kills Vemurafenib-resistant A375 melanoma cells by diminishing the levels of resistance-associated genes. **A** IC_50_ of Vemurafenib in Vemurafenib-sensitive A375 cell line. **B** IC_50_ of Vemurafenib in Vemurafenib-resistant A375 cell line. **C** Dose-dependent (0.5 µM, 1 µM, 2 µM, and 5 µM) effects of CST on cell viability of Vemurafenib-resistant A375 cells after 0, 24 and 48 h of treatment (*n* = 6). **D** Phase-contrast image of control versus CST-treated Vemurafenib-resistant A375. **E** Bar graph showing quantitative analysis of Trans well migration assay in Vemurafenib-resistant A375 cells (*n* = 3). **F** Cell viability of Vemurafenib-resistant A375 cells treated with Vemurafenib, CST and Vemurafenib along with CST and control for 24 h. **G** Volcano plot of differentially expressed genes upon CST treatment in A375 resistant cell (*n* = 4, each group) (**H**) Heatmap showing downregulated genes upon CST treatment with *p*adj < 0.05. **I** Heatmap of downregulated oncogenes with known link to resistance towards standard melanoma treatment including *WNT5A*, *KDM5B*, *PDGFRB*, *ID1*, *ID2*, *ID3*, *TWIST1*, *VAV3*, *FGFR3*, *SPARC*, *SOX2*, *SOX4*, *SREBP1*, and *MITF*. **J** Real-time PCR analysis of con and CST-treated Vemurafenib-resistant A375 cells. **K**–**M** Western blot of control and CST-treated A375 Vemurafenib-resistant cells. Cell viability assay graphs were analysed using 2-way ANOVA, followed by Dunnett’s multiple comparison test and Welch’s t test for analysis of the transwell migration data. Cell viability assay graph of Vemurafenib, CST treatment alone or in combination was analysed using 1-way ANOVA. *p ≤ 0.05, **p ≤ 0.01, and ***p ≤ 0.001.

Resistance to BRAF inhibitors such as Vemurafenib remains a major clinical challenge. Notably, the patient-derived melanoma line 156681 (detailed in Supplementary Table 1), isolated from a Vemurafenib-treated non-responder, displayed enhanced CST sensitivity with increased cell death as observed previously (Fig. 1). This prompted us to check CST’s anti-cancerous potential in Vemurafenib-resistant cells. To validate this observation, we generated a Vemurafenib-resistant A375 cell line. The IC₅₀ for resistant cells (1432 nM) was >6-fold higher than that of parental cells (228.9 nM) (Fig. 6A, B). CST treatment (0.5 µM, 1 µM, and 2 µM) markedly reduced viability of resistant A375 cells (Fig. 6C) and induced extensive cytopathic changes (Fig. 6D). Transwell migration assays confirmed diminished migration following CST exposure (Fig. 6E). To elucidate whether CST could overcome Vemurafenib resistance, we treated the resistant cells with Vemurafenib(2uM), CST (2 µM), their combination and control. After 48 h, Vemurafenib alone had little effect on cell viability, while CST alone and in combination with Vemurafenib led to a substantial decrease in cell viability of the A375 resistant cells, indicating that CST can reverse the acquired resistance (Fig. 6F). To further into the molecular mechanism of these observed effects, transcriptomics was performed in control and CST-treated resistant A375 cells.

Transcriptomic analysis of resistant cells treated with CST revealed extensive transcriptional reprogramming (Fig. 6G, H, Supplementary Fig. S5A, B) with 1990 upregulated and 1991 downregulated genes, including downregulation of *WNT5A* [46]*, PDGFRB* [47]*, ID1–3* [48–50]*, FGFR3* [51]*, SREBF1* [52]*, VAV3* [53]*, KDM5B* [54–56]*, TWIST1* [57]*, SOX2* [58]*, SOX4* [59]*, MITF* [60], and *SPARC* [61] (Fig. 6I) - genes associated with resistance and metastasis. Expression of genes *ID1*, *ID3*, *TWIST1* and *SPAR*C were further validated by real-time PCR analysis (Fig. 6J) and western blot (Fig. 6K–M, Supplementary Fig. S5C, D) The suppression of these genes that are actively involved in conferring resistance to known treatment regimens underscores the therapeutic potential of CST in overcoming treatment-resistant melanoma.

## Discussion

Melanoma incidence continues to rise worldwide, with an estimated 331,647 new cases and 58,645 deaths reported in 2022 (GLOBOCAN 2022) [62]. In the United States alone, over 104,000 new invasive melanoma cases are projected for 2025. Despite significant advances in targeted therapies and immune checkpoint inhibitors, tumor recurrence, metastatic progression, and therapeutic resistance remain major clinical challenges [63]. These limitations underscore the urgent need to identify novel therapeutic strategies that can suppress melanoma progression and overcome resistance to current treatments.

In this study, we provide the first evidence linking the CgA–derived peptide CST to melanoma pathobiology, identifying it as a previously unrecognized regulator of melanoma growth and progression. Peptide-based therapeutics remain relatively underexplored in melanoma [64–67], despite offering several advantages, including high biocompatibility, low immunogenicity, and the potential for rational structural optimization. CST has previously been implicated in regulating inflammation, oxidative stress, and metabolic homeostasis [16, 22], biological processes are increasingly recognized as critical determinants of tumor initiation and progression. These properties make CST an attractive candidate for investigating peptide-based therapeutic strategies targeting melanoma.

CST expression decreases with melanoma progression, suggesting that CST loss may facilitate tumor development. Restoration of CST in patient-derived melanoma cells elicited marked cytotoxicity and apoptosis in lines with distinct molecular features - K06184 (wild-type BRAF, brain metastasis), 156681 (BRAF^V600E, Vemurafenib-non-responder), and 128128 (treatment-naïve metastatic melanoma) - highlighting its broad anti-cancer efficacy across diverse genetic backgrounds.

Consistent with these findings, CST exerted potent anti-proliferative and anti-migratory effects. In both mouse (B16F10) and human (A375, SKMEL28) melanoma cell lines, CST treatment reduced cell viability, colony formation, and transwell migration. In vivo, CST administration attenuated tumor growth in B16F10 melanoma-bearing C57BL/6 mice, accompanied by reductions in Ki-67 and increases in TUNEL and cleaved caspase-3 signals, consistent with reduced proliferation and increased apoptosis. Importantly, the absence of changes in body weight or hepatic histology confirmed that CST treatment was well tolerated and systemically non-toxic.

To define CST-driven molecular alterations, transcriptomic analyses identified downregulated gene clusters enriched in pathways controlling extracellular-matrix organization, collagen metabolism, hypoxia adaptation, and epithelial-to-mesenchymal transition (EMT) - processes central to melanoma invasion and metastasis. CST significantly suppressed CCN2, LOXL2, DDIT4, and FN1, key drivers of ECM remodeling and angiogenesis, indicating that CST disrupts structural and metabolic programs sustaining tumor progression.

Although several CgA-derived peptides have been reported to influence angiogenesis [28, 68], the role of CST in tumor-associated angiogenesis has not previously been investigated. Our transcriptomic analyses revealed significant downregulation of pathways associated with hypoxia signaling and extracellular matrix remodeling following CST treatment. Hypoxia-driven transcriptional programs are well-established regulators of tumor angiogenesis and vascular remodeling in melanoma. The suppression of genes such as *CCN2*, *LOXL2*, and *DDIT4*, which are linked to ECM remodeling, hypoxia responses, and angiogenic signaling, suggests that CST may indirectly influence angiogenic processes within the tumor microenvironment. While direct measurements of tumor vascular density were beyond the scope of the present study, these transcriptional changes provide mechanistic evidence that CST may attenuate pro-angiogenic signaling networks associated with melanoma progression.

Given the prevalence of therapeutic resistance, particularly to BRAF/MEK inhibitors, we investigated the potential role of CST in Vemurafenib-resistant A375 cells. CST markedly reduced cell viability and migration in these resistant cells, accompanied by downregulation of multiple resistance-associated genes, including *FGFR3* [51, 69], *ID1/2/3* [48, 70], *PDGFRB*, *SREBF1* [52, 71], *and MITF* [72]. These genes are central mediators of adaptive survival signaling in BRAF^V600E melanoma, suggesting that CST not only restrains tumor growth but may also help overcome mechanisms underlying therapeutic resistance.

Overall, our findings demonstrate that CST suppresses melanoma growth and resistance while maintaining a favorable safety profile. Although CST has been reported to promote angiogenesis in certain physiological contexts [28, 68], our in vivo and transcriptomic data support a predominantly anti-proliferative and anti-tumorigenic role for CST in melanoma.

As CST emerges as a novel modulator of carcinogenesis, further mechanistic studies will be required to fully elucidate its molecular targets and signaling pathways. In parallel, peptidomimetic optimization of CST to improve stability, half-life, and tumor bioavailability may further enhance its therapeutic potential. Combination therapy paradigms integrating CST with BRAF/MEK inhibitors or immune checkpoint blockade also warrant investigation to determine whether synergistic anti-tumor effects can be achieved. Moreover, because CgA gives rise to multiple bioactive fragments - including PST, Vasostatin I/II, WE14, and Serpinin [14] - systematic studies examining their processing and functional interactions in melanoma may reveal complementary or cooperative anti-tumor mechanisms.

In conclusion, this study identifies catestatin as a previously unrecognized regulator of melanoma progression and provides compelling evidence supporting its anti-tumor activity in both treatment-naïve and drug-resistant melanoma models. By simultaneously targeting tumor growth, metastatic potential, and resistance-associated pathways, CST represents a promising foundation for the development of a new class of peptide-based therapeutics for melanoma.

## Supplementary information

Supplementary Figures

Supplementary Table

## Data Availability

Data supporting the conclusion of the current study is included in the manuscript and supplementary files. Bulk RNA sequencing data is available on GSE312638, and the codes used for RNA-Seq analysis are available on reasonable request. Uncropped images for the western blots are included in Supplementary Figures 6,7,8,9.
