# Supplementary material for: Catestatin peptide impedes melanoma progression and drug resistance by reprogramming oncogenic signaling pathways
Source: Oncogenesis. 2026 May 21;15(1):39. doi: 10.1038/s41389-026-00628-y (PMC13369845; doi:10.1038/s41389-026-00628-y)
Supplement: Supplementary file 1 — Supplementary Figures [file 41389_2026_628_MOESM1_ESM.pdf]

Catestatin peptide impedes melanoma progression and drug resistance by reprogramming oncogenic signaling pathways

Satadeepa Kal[1,3]*, Suborno Jati[2], Kechun Tang[1], Nicholas J.G. Webster[3,4], Angelo Corti[5], and Sushil K. Mahata[3,4]*

[1] Veterans Medical Research Foundation, San Diego, CA, USA
[2] Department of Neurosciences, University of California, San Diego, CA, USA
[3] Department of Medicine, University of California, San Diego, CA, USA
[4] VA San Diego Healthcare System, San Diego, CA, USA
[5] IRCCS San Raffaele Scientific Institute, San Raffaele Vita-Salute University, Milan, Italy

Running Title:

Catestatin impedes Melanoma progression

Keywords:

Catestatin, melanoma, peptide therapy, drug resistance reversal

Corresponding Authors:

Sushil K. Mahata, Ph.D.
Metabolic Physiology & Ultrastructural Biology Laboratory
Department of Medicine
University of California, San Diego (0732)
9500 Gilman Drive
La Jolla, CA 92093-0732, USA
Tel: (858) 552-8585 ext. 2637
Email: smahata@health.ucsd.edu

and

Satadeepa Kal, Ph.D.
Email: skal@health.ucsd.edu

Supplementary Figure 1. Anti-proliferative effects of CST on melanoma patient derived cell.

A.

Optical Density (490 nm)

Solid-phase polypeptide	Dilution (1/dil) 3000		9000		27000		0	
none (PBS)	0,053	0,051	0,047	0,047	0,045	0,045	0,043	0,046
$CgA_{1\text{-}439}$#6	0,076	0,074	0,058	0,055	0,05	0,047	0,045	0,043
$CgA_{1\text{-}439}$#5	0,107	0,104	0,064	0,065	0,052	0,052	0,045	0,043
His $CgA_{1\text{-}400}$	0,114	0,125	0,07	0,072	0,055	0,055	0,043	0,045
$CgA_{1\text{-}394}$	0,414	0,421	0,167	0,158	0,109	0,103	0,045	0,043
His $CgA_{1\text{-}373}$	2,974	2,873	2,017	1,992	1,081	1,06	0,044	0,044
$CgA_{1\text{-}372}$	2,88	2,943	1,93	1,975	1,01	0,994	0,046	0,046
$CgA_{1\text{-}338}$	0,074	0,071	0,059	0,056	0,048	0,047	0,044	0,043
$CgA_{352\text{-}373}$	2,487	2,479	1,519	1,518	0,749	0,768	0,036	0,046
$CgA_{352\text{-}372}$	2,766	2,72	1,802	1,786	0,927	0,903	0,044	0,044
$CgA_{367\text{-}373}$	0,085	0,085	0,063	0,06	0,056	0,058	0,047	0,046
$CgA_{367\text{-}372}$	0,112	0,105	0,066	0,06	0,054	0,05	0,046	0,045
Vasostatin	0,074	0,071	0,056	0,053	0,048	0,048	0,044	0,043

anti 352-372 (VFA/09-C2)

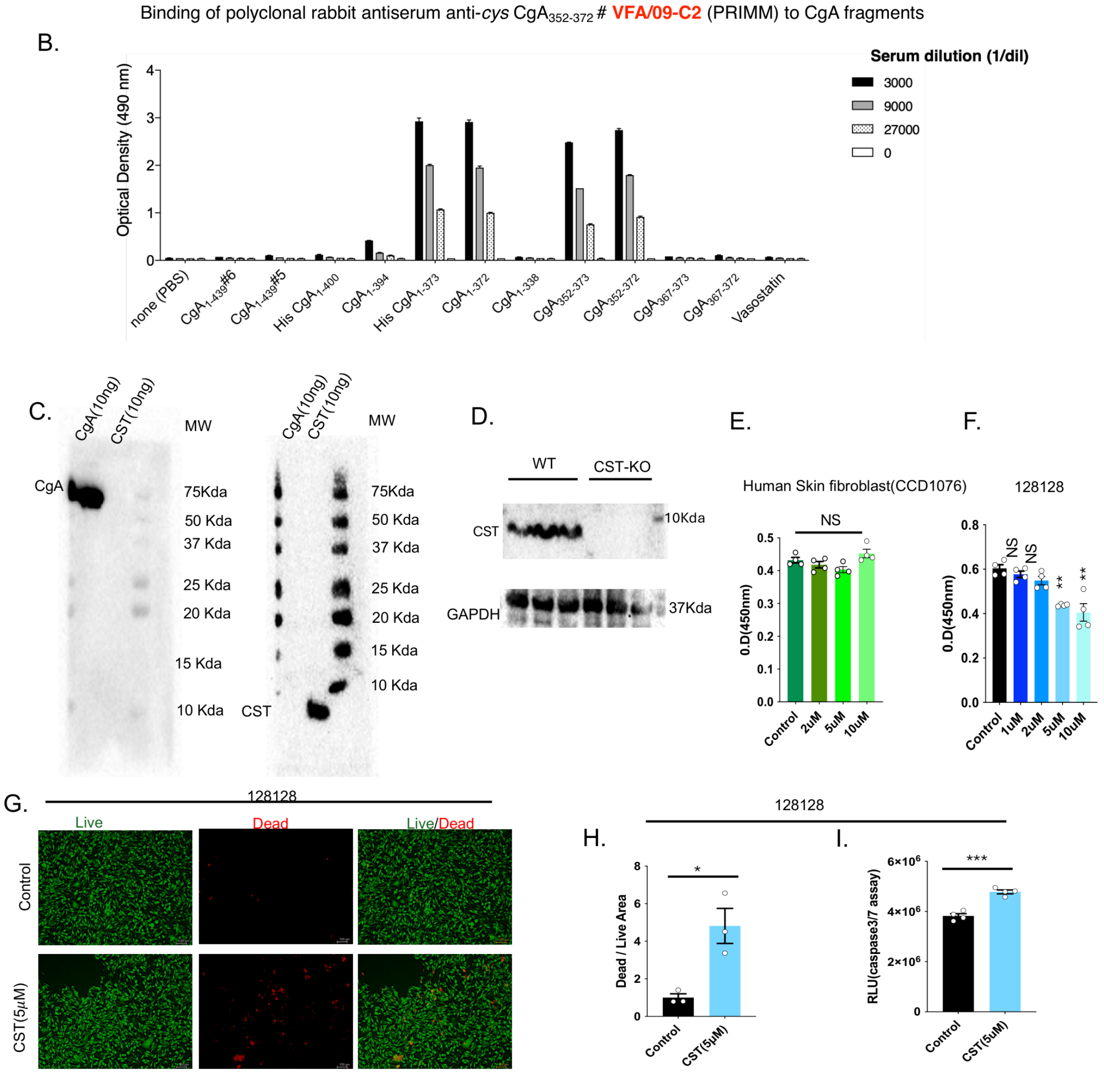

Validation and specificity of the CST antibody. **(A&B)** ELISA-based epitope mapping of the CST antiserum showing antibody reactivity toward multiple CgA fragments across increasing antibody concentrations. The antibody selectively

recognizes fragments containing the CST region ($CgA_{352–372/373}$), while showing no detectable binding to unrelated CgA regions or larger fragments lacking accessible CST epitopes. Quantitative binding curves demonstrate concentration-dependent antibody reactivity. (**C&D**) Immunoblot validation of antibody specificity. Purified CgA protein and CST peptide were probed with anti-CgA and anti-CST antibodies to assess cross-reactivity. Additional immunoblotting of heart tissue lysates from wild-type and CST-knockout mice confirms selective detection of the CST peptide without cross-reactivity to full-length CgA. (**E&F**). Bar graph showing cell viability assay on Human skin fibroblast (CCD1076) and patient derived cell 128128 for 120 hours in response to different concentration of CST (1 μM, 2 μM, 5 μM, and 10 μM) versus vehicle control. (**G)** Micrographs showing green-stained (with calcein AM) live 128128 cells and red-stained (with EthD-III) dead 128128 cells after treatments with CST (5 μM) versus control. Images were captured in Keyence microscope at a magnification of 10X. Scale bar: 100 μm. (**H)** Bar graph showing quantitative analysis of dead/live areas upon CST treatment in 128128 cells. (**I**) Bar graph showing relative light units from caspase 3/7 assay after treatment of 128128 cells with CST or vehicle control. Data were presented as Mean ± SEM and analyzed by Welch's t-test (**D&E**). *$p \leq 0.05$, **$p \leq 0.01$, and ***$p \leq 0.001$.

Supplementary Figure 2. Melanoma progression inhibition in human melanoma cells upon CST treatment

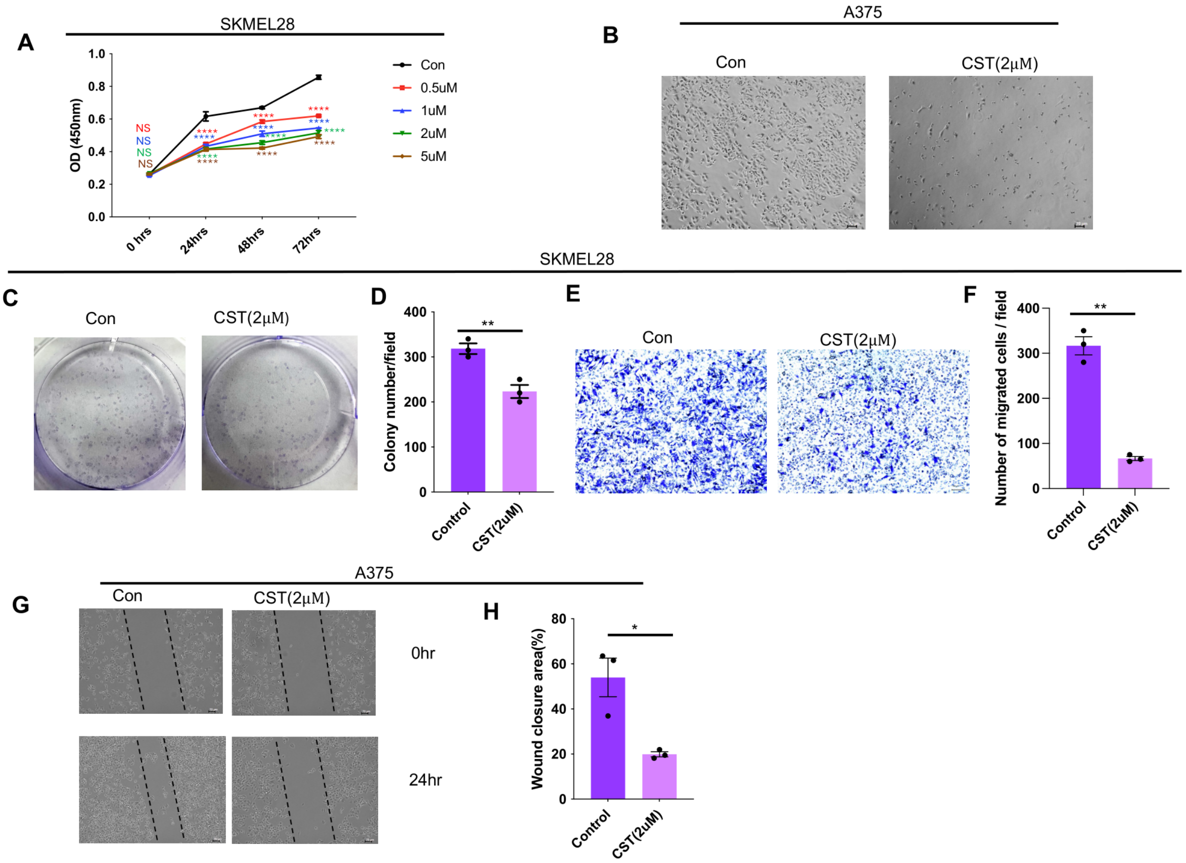

(A) Cell viability assay of SKMEL28 cell line by CCK-8 assay using different concentrations of CST (0.5 μM, 1 μM, 2 μM, and 5 μM) versus vehicle control for 0, 24 ,48 and 72 hours. Phase contrast imaging of Control versus CST (2 μM)-treated A375 human melanoma cells for 24 hours. Images were taken at a magnification of 20X. scale bar: 70 μm. **(B&C)** Colony formation assay in SKMEL28 cells after treatments with CST versus control and its quantitative analysis. Brightfield images were captured in of Keyence microscope at a magnification of 10X. Scale bar: 100 μm. (**D&E)** Transwell migration assay in control and CST treated SKMEL28 cells and its quantitative analysis. (**F&G)** Wound healing assay in A375 cells after treatments with CST versus vehicle control for 24 hours and its quantitative analysis. Two-way ANOVA followed by Dunnett's multiple comparison test was used to analyze cell viability assay data. Wound healing assay was presented as Mean ± SEM and analyzed by Welch's t-test (**C, E&G**). *$p \leq 0.05$, **$p \leq 0.01$, and ***$p \leq 0.001$.

Supplementary Figure 3. *In vivo* melanoma tumor effects upon CST treatment.

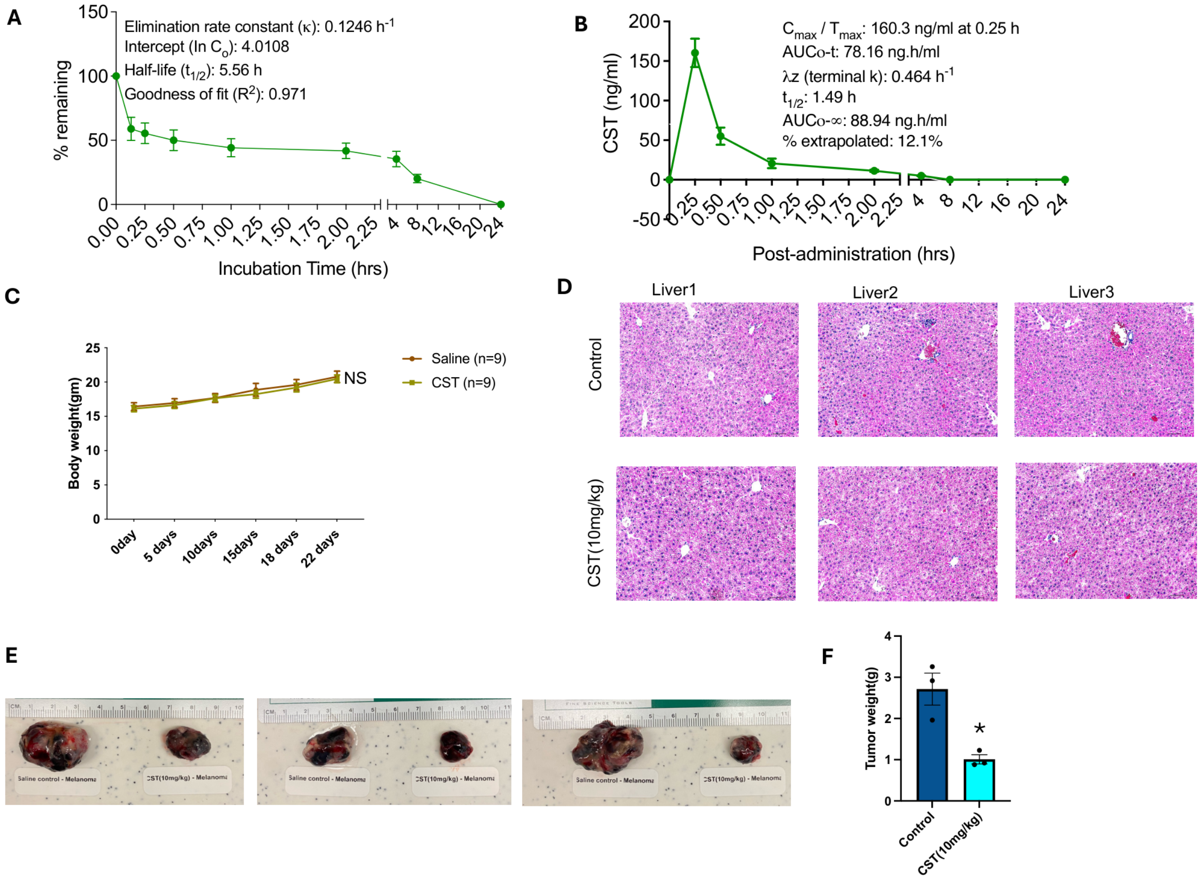

(**A**) *In vitro* plasma stability of CST monitored till 24 hours. (**B**) In vivo plasma pharmacokinetics of CST till 24 hours. (**C**) Regulation of body weight by CST (10mg/kg) versus vehicle control in mice bearing B16F10 derived tumors. Data were presented as Mean ± SEM and analyzed by 2-way ANOVA with Sidak's multiple comparison test. **(D)** Histological micrographs of hematoxylin and eosin stained liver sections in mice after treatments with CST versus vehicle control. (**E&F**) Tumor size and weight of Control and CST-treated tumor after intratumoral treatment of CST (10mg/kg) for 3 days a week (Monday, Wednesday, Friday) for 15 days.

Supplementary Figure 4. Molecular mechanism of CST treatment in melanoma tumor and A375 cell line

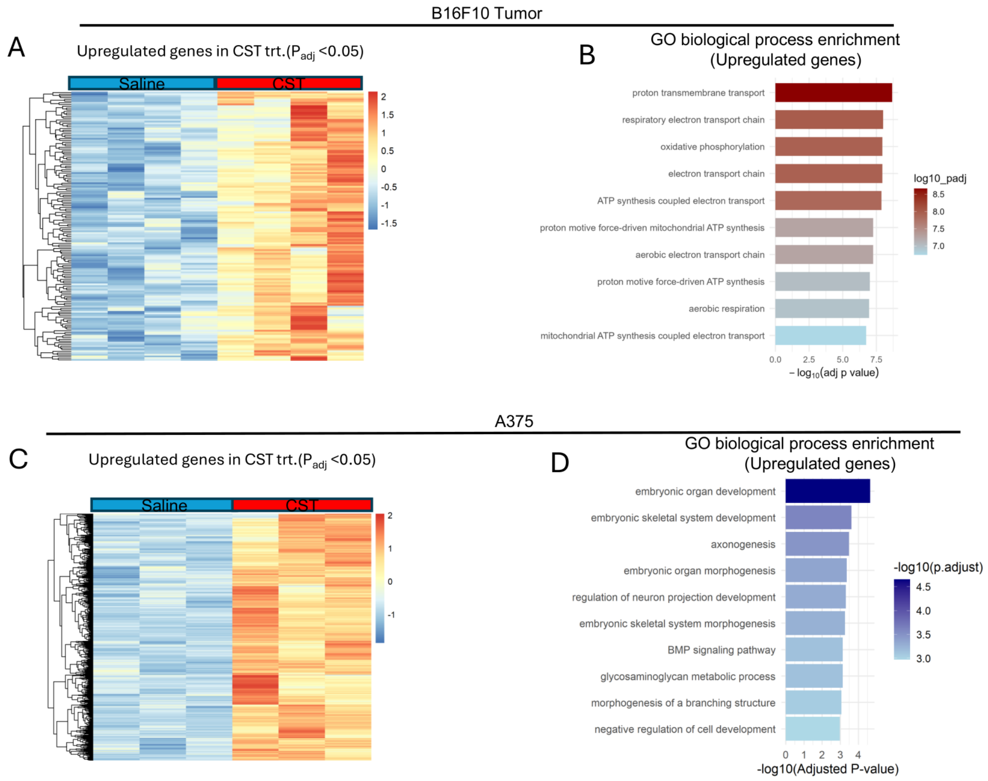

(A) Heatmap showing upregulated genes upon CST treatment with padj <0.05 in B16F10 tumor (n=4, each group) **(B)** GO analysis showing enriched upregulated pathways in B16F10 tumor in response to CST treatment. **(C)** Heatmap showing upregulated genes in A375 cells after treatments with CST with padj<0.05 (n=3, each group) **(D)** GO analysis showing enriched upregulated pathways in A375 cells upon CST treatment.

Supplementary Figure 5. Molecular mechanism of CST treatment in Vemurafenib resistant melanoma cell A375

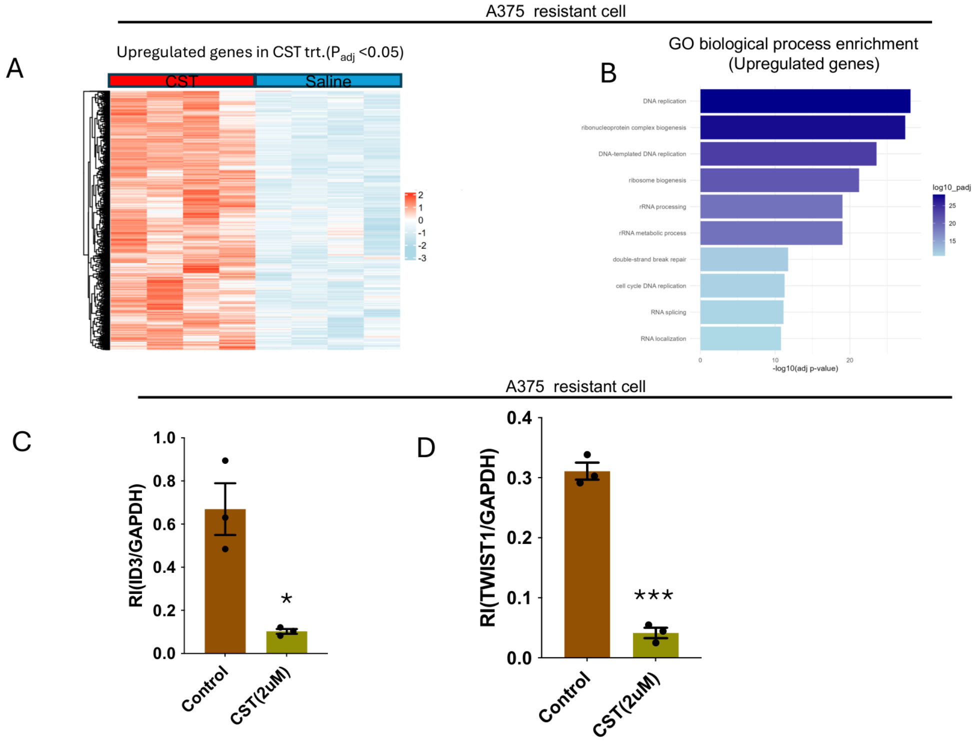

(A) Heatmap showing upregulated genes in A375 resistant cells upon CST treatment with padj<0.05 (n=4, each group) (**B**) GO analysis showing enriched upregulated pathways in A375 resistant cells in response to CST treatment. (**C&D**) Quantification of ID3 and TWIST1 protein in Con and CST treated A375 vemurafenib resistant cells.
