# Supplementary material for: Catestatin peptide impedes melanoma progression and drug resistance by reprogramming oncogenic signaling pathways
Source: Oncogenesis. 2026 May 21;15(1):39. doi: 10.1038/s41389-026-00628-y (PMC13369845; doi:10.1038/s41389-026-00628-y)
Supplement: Supplementary file 2 — Supplementary Table [file 41389_2026_628_MOESM2_ESM.pdf]

Catestatin peptide impedes melanoma progression and drug resistance by reprogramming oncogenic signaling pathways

Satadeepa Kal[1,3]*, Suborno Jati[2], Kechun Tang[1], Nicholas J.G. Webster[3,4], Angelo Corti[5], and Sushil K. Mahata[3,4]*

[1] Veterans Medical Research Foundation, San Diego, CA, USA
[2] Department of Neurosciences, University of California, San Diego, CA, USA
[3] Department of Medicine, University of California, San Diego, CA, USA
[4] VA San Diego Healthcare System, San Diego, CA, USA
[5] IRCCS San Raffaele Scientific Institute, San Raffaele Vita-Salute University, Milan, Italy

Running Title:

Catestatin impedes Melanoma progression

Keywords:

Catestatin, melanoma, peptide therapy, drug resistance reversal

Corresponding Authors:

Sushil K. Mahata, Ph.D.
Metabolic Physiology & Ultrastructural Biology Laboratory
Department of Medicine
University of California, San Diego (0732)
9500 Gilman Drive
La Jolla, CA 92093-0732, USA
Tel: (858) 552-8585 ext. 2637
Email: smahata@health.ucsd.edu

and

Satadeepa Kal, Ph.D.
Email: skal@health.ucsd.edu

Supplementary Table 1: Details of melanoma patient-derived cells

Patient ID	Sex	Tissue type	Mutation	Treatment regime	Metastasis(yes/No-location if known)
K06184	Male	Brain resection, at diagnosis found at right calf	BRAF wildtype at diagnosis, c-kit not detected	Ipilimumab, Radiation, Pembrolizumab, Radiation again	Yes (Brain, right thigh)
156681	Female	Left forearm resection	V600E BRAF	TVEC, Ipilimumab, Decitabine, Vemurafenib. Only the left forearm did not respond	Yes
128128	Male	Lymph node resection	Not known	Treatment naive	Yes (lymph node)

Supplementary Table 2: Significance values of genes in primary vs metastasis of human melanoma TCGA dataset

Gene Name	Statistical significance (primary vs. metastasis)
CTGF (CCN2)	7.12750000020357E-07
DDIT4	1.057340E-03
PDGFRB	1.28280000000203E-06
LOXL2	1.159810E-01

Supplementary Table 3: Sequence of primers used for the study

Gene Name	Primer sequence for qRT-PCR
Human *ID1*	FP- 5’ GTTGGAGCTGAACTCGGAATCC 3’<br>RP- 5’ ACACAAGATGCGATCGTCCGCA 3’
Human *ID3*	FP- 5’ CAGCTTAGCCAGGTGGAAATCC 3’<br>RP- 5’ GTCGTTGGAGATGACAAGTTCCG 3’
Human *TWIST1*	FP- 5’ GCCAGGTACATCGACTTCCTCT 3’<br>RP- 5’ TCCATCCTCCAGACCGAGAAGG 3’
Human *SPARC*	FP- 5’ TGCCTGATGAGACAGAGGTGGT 3’<br>RP- 5’ CTTCGGTTTCCTCTGCACCATC 3’
